# Conditions and Regulation of Mixed Culture to Promote *Shiraia bambusicola* and *Phoma* sp. BZJ6 for Laccase Production

**DOI:** 10.1038/s41598-017-17895-w

**Published:** 2017-12-19

**Authors:** Wen Du, Chunlong Sun, Jun Wang, Wenjun Xie, Baoqin Wang, Xuehong Liu, Yumiao Zhang, Yanhui Fan

**Affiliations:** 1 School of bioengineering, Binzhou University, Binzhou, China; 2 Shandong provincial key laboratory of eco-environmental science for Yellow River Delta, Binzhou University, Binzhou, China

## Abstract

Mixing cultures induces the biosynthesis of laccase in mixed cells, produces signal molecules, and regulates the production of mixed-cell metabolites. The fungal strain, which promotes laccase production, has been isolated and screened from the host bamboos of endophytic fungi and identified as *Phoma* sp. BZJ6. When the culture medium is mainly composed of soluble starch, yeast extract, and *Phoma* sp., the laccase output can reach 4,680 U/L. Nitric oxide (NO) and reactive oxygen species (ROS) were found to promote the regulation of laccase synthesis. Plasma membrane NAD(P)H oxidase inhibitors and NO-specific quenchers can inhibit not only the accumulation of ROS induced and NO synthesis but also the biosynthesis of laccase. The results indicate that the accumulation of superoxide anion radical (O_2_^−^) and hydrogen peroxide (H_2_O_2_) induced by the mixed culture was partially dependent on NO. The mixed culture can also reduce the biomass, increase the synthesis of total phenolics and flavonoids, and enhance the activity of phenylalanine ammonia-lyase and chalcone isomerase. This phenomenon is probably the result of the activated phenylpropanoids–flavonoid pathway. Results confirmed that the mixture culture is advantageous for laccase production and revealed that NO, O_2_^−^, and H_2_O_2_ are necessary signal molecules to induce laccase synthesis.

## Introduction

Laccases are copper-containing polyphenol oxidases and are homologous with ascorbate oxidase and with mammalian plasma protein ceruloplasmin, which belong to the blue multi-copper oxidase family and have several similarities in structure and functionality^1^. Laccases play vital roles in the natural carbon cycle and facilitate direct reduction of molecular oxygen to water^2^. Even in the absence of H_2_O_2_ and of other secondary metabolites, the substrate may be directly oxidized by laccases as long as dissolved oxygen exists^3^. Laccases were originally found in a peculiar sap of *Rhus vemicifera* and extensively exist in nature, specifically in plants, fungi, insects, and bacteria. Laccase-producing fungi are mainly present in Basidiomycota, Ascomycota, Deuteromycota, and other divisions, and are especially widely distributed in white rot fungi^4^. Laccases have great practical importance and potential in biosynthesis, bio-scavenging of toxic compounds^5^, bioassay and immunoassay^6^, paper-making, biosensor^7^, biocatalysis^8^, new energy, and food processing^9^. As a result, laccases have attracted considerable research interest in recent years.

*Shiraia bambusicola* P. Henn. is a fungus that grows in bamboo poles and is mainly distributed in Japan, Sri Lanka, and in the Zhejiang, Jiangsu, Anhui, and Guizhou Provinces in China^10^. *S*. *bambusicola* is featured by host specificity and grows only in several species of bamboo, primarily in *Brachystachyum densiflorum* (Rendle) Keng and *Pleioblastus amarus* (Keng) Keng f^11^. *S*. *bambusicola* is subordinate to Shiraiaceae, which is a family of Pleosporales under Dothideomycetes in the Ascomycota. This species is a key fungus for the production of laccase^12,13^. The laccase is widely distributed in nature; however, only a few strains can contribute to the mass production of laccase, although these strains can be screened out from nature or can be modified. Methods to increase the laccase yield affect the booming laccase industry, and many researchers are committed to optimizing the fermentation media and culture conditions for different microbes. Laccases are composed of the constitutive and inducible types, which are affected by many fermentation factors, including carbon and nitrogen sources, inducers, pH, ventilation, temperature, culture time, and so on^14,15^. Another focus of research attention is the screening of new microbes with high laccase yield. Strains that produce a large number of enzymes can be determined through strain screening and gene cloning of laccase-producing fungi. Heterologous laccase expression can be up-regulated by using genetic engineering techniques, which is a simple method for improving the laccase yield; however, most fungal laccases are not efficient for heterologous expression systems, thereby limiting their application^15–17^. In recent years, scholars have studied the fermentation of compound microbes to increase the laccase yield. The mixed fermentation of *Coprinopsis cinerea* Okayama^7^ (#130) and *Gongronella* sp. w5 might effectively improve the laccase yield, probably because silent laccase genes were activated, and the resultant laccases might have produced certain resistance to adverse environments^18^. The optimal combination method was selected after *Polyporellus picipes*, *Pleurotus ostreatus*, and *Pseudotrametes gibbosa* were cultured in different white rot fungi, and the laccase yield peaked at 14 days after fermentation^19^. In addition, the mixed fermentation of white rot fungi *Dichomitus squalens* and *Ceriporiopsis subvermispora* also increased the laccase yield^20^. The co-culture of *Trametes versicolor* and *Candida* sp. HSD07A led to glucose starvation, thereby increasing the laccase yield^21^. However, during the co-culture, numerous related topics, such as the isolation of co-existing fungi, enhancement of laccase production, and identification of regulatory mechanism to increase production, are worthy of investigation.

In this paper, the strains, which can promote laccase production have been identified and screened from the endophytic fungi in the host bamboos of *S*. *bambusicola*, and the carbon source, nitrogen source, and additive amount of endophytic fungi were selected to determine the optimum conditions for mixed fermentation to induce laccase synthesis. The mixed cells (*S*. *bambusicola* and *Phoma* sp. BZJ6) were used to study the signal molecules required for laccase synthesis induced by mixed fermentation. Moreover, the influence of mixed fermentation on the biomass of mixed cells, phenolics compounds, and enzymatic activity were explored. Few studies have focused on the use of endophytic fungi to screen mixed strains or on the activating effect of regulatory mechanism of mixed culture on the two signal molecules. Therefore, this study may probably accelerate the development of the laccase fermentation industry. The results will be of great significance in understanding the regulatory mechanism of mixed culture when it induces the synthesis of fungal metabolites.

## Results

### Separation, screening, and identification of endophytic fungi promoted the mixed cells to produce laccase

By taking the special advantages of environmental factors, the task group selected *B*. *densiflorum* growing *S*. *bambusicola* as the material for bio-separation in the experiment. Endophytic fungi were isolated from such materials, and those likely promoting the laccase yield of *S*. *bambusicola* were screened by liquid culture. A total of 36 strains of endophytic fungi were isolated from *B*. *densiflorum*, the host of *S*. *bambusicola*. However, not all endophytic fungi might increase the laccase yield in the mixed culture with *S*. *bambusicola*. Moreover, liquid culture was used to determine whether the mixed culture will promote the synthesis of laccases. The strain BZJ6 that had the best promoting effect was screened from the 36 strains of endophytic fungi. The laccase yield increased 4.3-fold in the mixed culture of *S*. *bambusicola*, and the strain BZJ6 suggested the effective method to increase the laccase yield.

The strain BZJ6 in potato dextrose agar (PDA) was cultured for 3–4 days at 26 °C, with the colony diameter up to 3.8 cm, and the mycelia were transparent, parted, and ramified, with widths of 0.9–1.7 or 3.0–6.2 (8.3) μm. The colony surface at the initial stage was flocculent or loosely fluffy, having white and light gray protrusions. The substrate mycelia were fine, closed, and dark brown, and formed stratified and concentric circular colonies of the same color depth. No conidial fructification occurred after culturing for 14 days.

**Figure 1:**
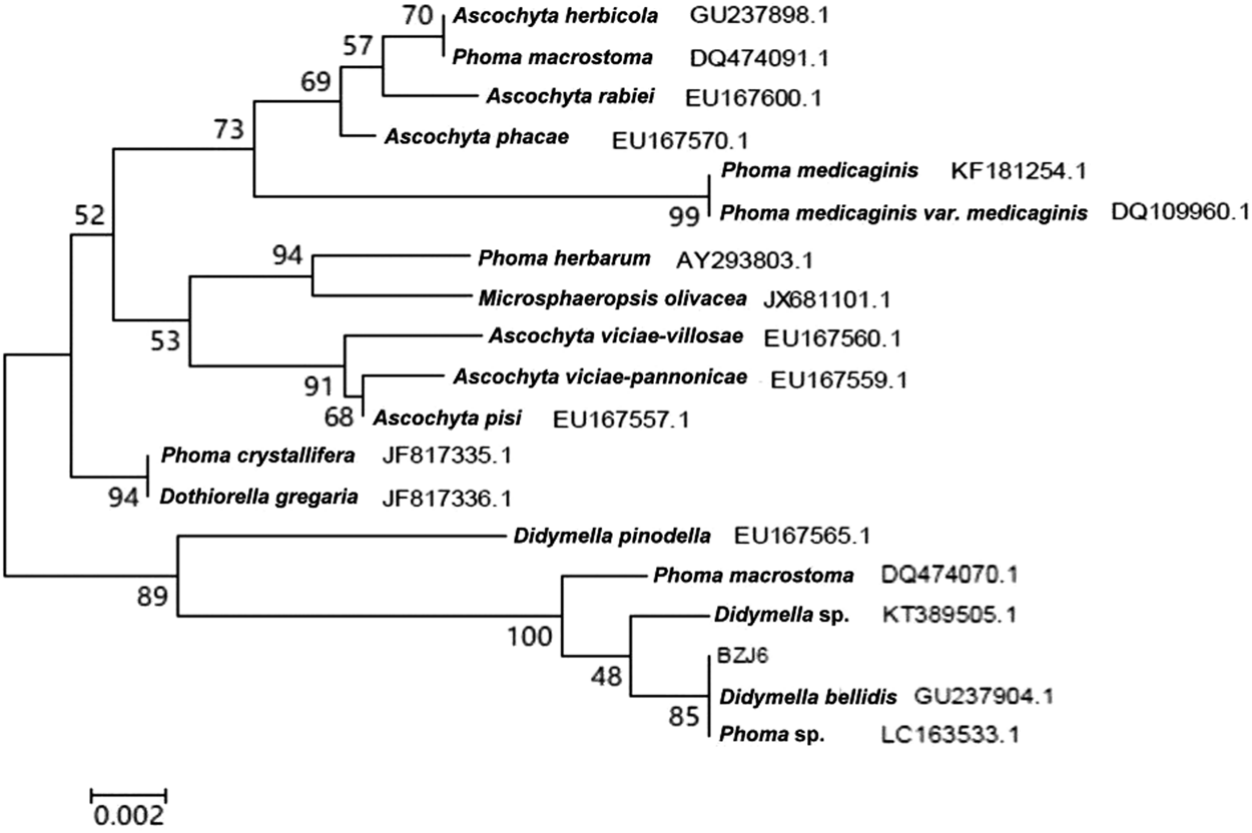
Neighbor-joining phylogenetic tree based on analysis of ITS1-5.8S-ITS2 rDNA sequences of *phoma* sp. strain BZJ6 and some related species from GenBank. Bootstrap values based on 1,000 replicates are given above the branches.

The fungi, in which the internal transcribed spacer (ITS) regions rapidly evolved, are polymorphic and suitable for the identification of those strains in closer phylogenetic relationships^22^. In the present experiment, the sequences of the ITS region were obtained for identifying BZJ6. The ITS sequences of BZJ6 were entered into the Genbank as ‘MF314176’. Using Mega 5.2, a phylogenetic tree was established by Neighbor–Joining method. As analyzed and observed, BZJ6 and some *Phoma* fungi in the GenBank might be well mixed in an evolutionary branch, with the support rate of 100%, and their ITS rDNA sequences were highly homologous in closer phylogenetic relationships (Fig. 1). As finally identified, BZJ6 belonged to *Phoma*, and thus, was designated as *Phoma* sp. BZJ6.

### Mass production of laccases

**Figure 2:**
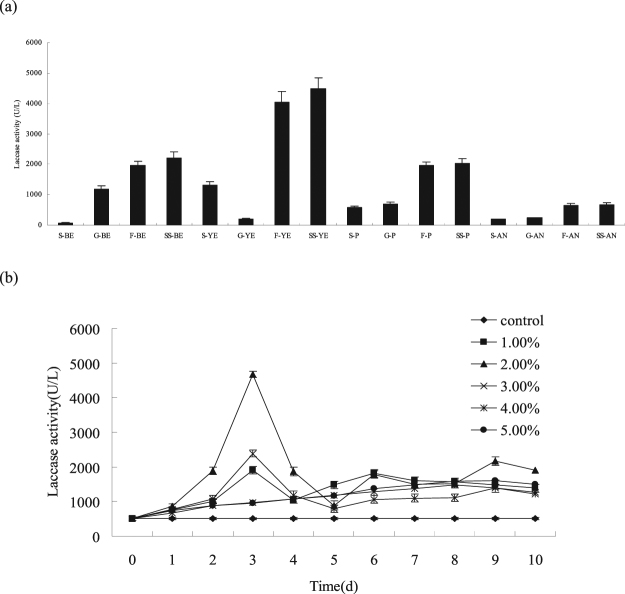
Effects of carbon, nitrogen sources (**a**) and inoculation volume of *Phoma* sp. BZJ6 (**b**) on laccase production by *S*. *bambusicola* in mixed culture with *Phoma* sp. BZJ6. Carbon sources: Sucrose (S), Glucose (**G**), Fructose (**F**), Soluble starch (SS). Nitrogen sources: Beef extract (BE), Yeast extract (YE), Peptone (**P**), Ammonium nitrate (AN).

After the carbon and nitrogen sources and the inoculation volume of *Phoma* sp. BZJ6 were optimized, the highest laccase yield was achieved in the mixed culture of *S*. *bambusicola* and *Phoma* sp. BZJ6. The optimal materials for laccase production, including soluble starch, yeast extract, and 2.0% (v/v) *Phoma* sp. BZJ6, were found in the test (Fig. 2). The laccase yield of mixed cells increased largely three days after providing the mixed culture with 2.0% (v/v) *Phoma* sp. BZJ6 and reached the maximum of 4680 ± 119 U/L (Fig. 2b). By contrast, the laccase yield during the pure culture of BZJ6 in flasks containing Czapek’s medium was only 66 ± 7 U/L, whereas the laccase yield was only 487 ± 11 U/L during the pure culture of *S*. *bambusicola* in flasks containing Czapek’s medium. The laccase yield in the non-mixed culture of *S*. *bambusicola* and *Phoma* sp. BZJ6 was only 510 ± 14 U/L under the above circumstances. The laccase activity in the co-culture system is 9.18 times higher than that in the pure culture, which means that the mixed culture may induce *Phoma* sp. BZJ6 and *S*. *bambusicola* to produce laccase, but the mechanism of increasing the laccase yield needs further study.

### Nitric oxide (NO) in the biosynthesis of laccases induced by mixed culture

**Figure 3:**
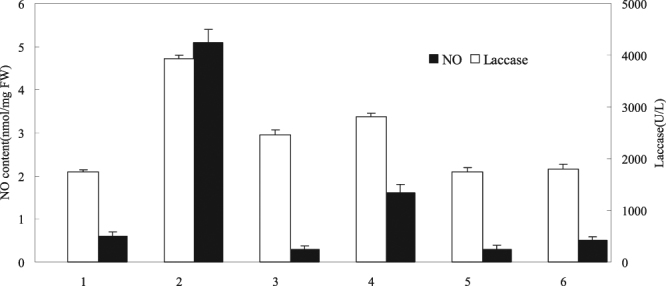
NO mediated the mixed culture to induce the synthesis of laccase. The experimental material was mixed with 2.0% (v/v) *Phoma* sp. BZJ6 with *S*. *bambusicola* for 3 days. The inhibitor was added 20 min before the addition of the mixed culture. NO content and laccase yield were measured at 2 and 4 days after mixed culture was added. The control group was *S*. *bambusicola* and *Phoma* sp. BZJ6 cultured individually, the culture conditions were the same as those of the mixed culture. The results shown are the average of three independent experiments. 1, control; 2, mixed culture; 3, mixed culture + cPITO (0.5 mmol/L); 4, mixed culture + AG (0.5 mmol/L); 5, control + cPITO (0.5 mmol/L); 6, control + AG (0.5 mmol/L).

The degree of induction in the mixed culture was reported to depend on the types and concentrations of mixed strains and on the cell growth stage while mixing^23^. The three-day culture medium of the *S*. *bambusicola* cell suspension was added with 0.5 mmol/L 2,4-carboxyphenyl-4,4,5,5-tetramethylimidazoline-1-oxyl-3-oxide (cPITO), S,S′-1,3-phenylene-bis(1,2-eth-anediyl)-bisisothiourea (PBITU), and 2.0% (v/v) *Phoma* sp. BZJ6 to produce the mixed culture. cPTIO (NO quencher) and PBITU (nitric oxide synthase inhibitor) were added 20 min before mixing the culture. The control group was *S*. *bambusicola*, and *Phoma* sp. BZJ6 was cultured individually; the culture conditions were the same as those of the mixed culture. The experimental results showed that the NO content increased 8.5-fold after the mixed culture of *S*. *bambusicola* and *Phoma* sp. BZJ6, which may induce the synthesis of NO, by comparing with those of the control group (Fig. 3). Previous studies showed that NO is a signaling molecule for biological stress factors, such as pathogenic microbes and fungal elicitors, to induce the synthesis of secondary metabolites in cells^24^. To explore the role of NO in the biosynthesis of laccases induced by the mixed culture, the effects of cPTIO on the NO content in mixed cells, and the accumulation of laccases were determined. The experimental results suggested that cPTIO may not only inhibit the NO synthesis induced by the mixed culture but also block a part of the inducing impact of the mixed culture on the accumulation of laccases in cells. cPTIO does not significantly affect the synthesis of laccases in mixed cells. The experimental results also indicated that NO might be a signaling molecule for the synthesizing laccases in cells induced by the mixed culture. Furthermore, the results in Fig. 3 showed that the nitric oxide synthase (NOS) inhibitor PBITU may also inhibit the inducing effect of the mixed culture on the synthesis of laccases in cells, further validating the above conclusions.

As shown in Fig. 3, NO is a signaling molecule for the synthesis of laccases in cells induced by the mixed culture, that is, the mixed culture may promote the laccase biosynthesis, relying upon the NO signaling pathway. However, the data in Fig. 3 show that even if the production of NO in cells was completely quenched using cPTIO, the mixed culture might still promote the synthesis of laccases, suggesting that NO was not perhaps the sole signaling pathway by which the mixed culture mediated the synthesis of laccases.

### Reactive oxygen species (ROS) were involved in the influence of mixed culture on laccase biosynthesis

**Figure 4:**
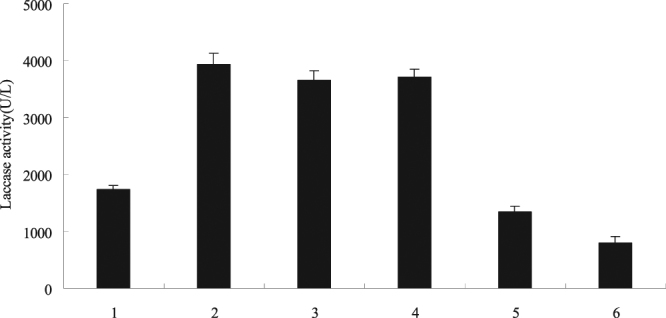
ROS mediated the mixed culture to induce the synthesis of laccase. The experimental material was mixed with 2.0% (v/v) *Phoma* sp. BZJ6 with *S*. *bambusicola* for 3 days. The inhibitor was added 20 min before the addition of the mixed culture. Laccase yield were measured at 4 days after mixed culture was added. The control group was *S*. *bambusicola* and *Phoma* sp. BZJ6 cultured individually, the culture conditions were the same as those of the mixed culture. The results shown are the average of three independent experiments. 1, control; 2, mixed culture; 3, mixed culture + glycerol (10 mmol/L); 4, mixed culture + histidine (10 mmol/L); 5, mixed culture + SOD (200 units/mL); 6, mixed culture + CAT (200 units/mL).

Under mixed culture and other stress conditions, one of the early reactions of cells was oxidative burst^25,26^. Oxidative burst produces various reactive oxygen intermediates^27^. To investigate the influence of the mixed culture and other signal molecules during laccase synthesis in cells and to explore the role of hydroxyl radicals, hydrogen peroxide (H_2_O_2_), superoxide anion radical (O_2_^−^), and singlet oxygen in mixed culture, various active oxygen scavengers and quenching agents were added to the mixture. The effects of hydroxyl radical, H_2_O_2_, O_2_^−^, and singlet oxygen on laccase synthesis were studied; for instance, glycerol can be used as a hydroxyl radical quencher, histidine as a singlet oxygen quencher, superoxide dismutase (SOD) as O_2_^−^ quencher, and catalase (CAT) as H_2_O_2_ quencher of^28–30^. As shown in Fig. 4, CAT and SOD can significantly reduce the yield of laccase in the mixed culture. The effects of glycerol and histidine on laccase production were not significant. Results showed that the mixed culture induced the oxidative burst and produced ROS. Furthermore, the mixed culture was closely related to the amount of H_2_O_2_ and O_2_^−^.

**Figure 5:**
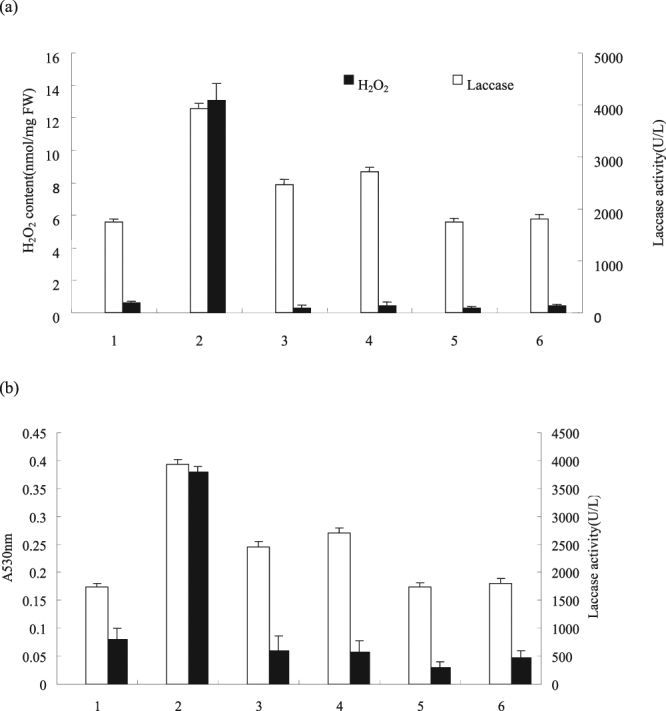
H_2_O_2_ (**a**) and (**b**) mediated the mixed culture to induce the synthesis of laccase. The experimental material was mixed with 2.0% (v/v) *Phoma* sp. BZJ6 with *S*. *bambusicola* for 3 days. The inhibitor was added 20 min before the addition of the mixed culture. Laccase yield were measured at 4 days after mixed culture was added. The control group was *S*. *bambusicola* and *Phoma* sp. BZJ6 cultured individually, the culture conditions were the same as those of the mixed culture. The results shown are the average of three independent experiments. (**a**) 1, control; 2, mixed culture; 3, mixed culture + DPI (10 mmol/L); 4, mixed culture + CAT (10 mmol/L); 5, control + DPI (0.5 mmol/L); 6, control + CAT (0.5 mmol/L). (b): 1, control; 2, mixed culture; 3, mixed culture + DPI (10 mmol/L); 4, mixed culture + SOD (10 mmol/L); 5, control + DPI (0.5 mmol/L); 6, control + SOD (0.5 mmol/L).

To explore other signal molecules and pathways that induced laccase synthesis in cells in the mediated mixed culture, the effects of the mixed culture on the content of H_2_O_2_ and O_2_^−^ in cells were investigated, and the effects of H_2_O_2_ and O_2_^−^ inhibitors on the synthesis and accumulation of laccase in cells were examined. Figure 5 shows that the mixed culture rapidly induced the increase of H_2_O_2_ and O_2_^−^ in cells. Further experimental results showed that superoxide dismutase (SOD), catalase (CAT), and plasma membrane NAD(P)H oxidase inhibitor diphenylene iodonium (DPI) not only inhibited the production of H_2_O_2_ and O_2_^−^ in the mixed culture but also inhibited the promoting effect of the mixed culture on laccase synthesis in cells. The above experimental results indicated that H_2_O_2_ and O_2_^−^ were also involved in the accumulation of necessary signal molecules during laccase synthesis in cells in the mixed culture.

### Partial dependence of NO on the oxidative burst of mixed culture cells

**Figure 6:**
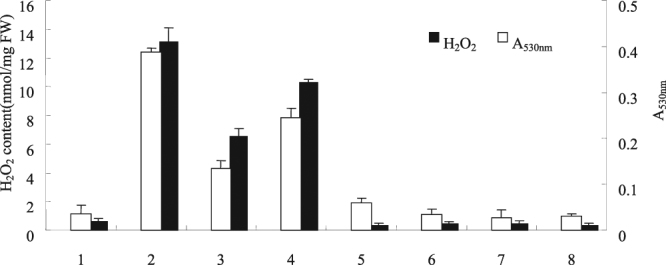
Inhibitors mediated the mixed culture to induce the synthesis of oxidative burst. The experimental material was mixed with 2.0% (v/v) *Phoma* sp. BZJ6 with *S*. *bambusicola* for 3 days. H_2_O_2_ content and A_530nm_ were measured at 2 days after mixed culture was added. The control group was *S*. *bambusicola* and *Phoma* sp. BZJ6 cultured individually, the culture conditions were the same as those of the mixed culture. The results shown are the average of three independent experiments. 1, control; 2, mixed culture; 3, mixed culture + cPITO (0.5 mmol/L); 4, mixed culture + PBITU (0.5 mmol/L); 5, mixed culture + DPI (0.5 mmol/L); 6, control + cPITO (0.5 mmol/L); 7, control + PBITU (0.5 mmol/L); 8, control + DPI (0.5 mmol/L).

The mixed culture induced NO and oxidative burst of cells (Figs 3–5). To study the relationship between the oxidative burst and NO accumulation in the mixed culture cells, cPITO and PBITU were added to *S*. *bambusicola* 20 min before the cells were mixed and cultured. The effects of NO quenchers and NOS inhibitors on the accumulation of H_2_O_2_ and O_2_^−^ in the mixed culture cells were investigated. Results showed that cPITO and PBITU inhibited the induction of H_2_O_2_ and O_2_^−^ in the mixed culture cells. Thus, Fig. 6 shows that the accumulation of oxidative burst in the mixed culture and the accumulation of O_2_^−^ and H_2_O_2_ were at least partially dependent on NO.

To explore other signaling molecular pathways by which the mixed culture mediated the synthesis of laccases in cells, the effects of the mixed culture on the H_2_O_2_ content in cells were determined, and those of the H_2_O_2_ inhibitor on the synthesis of laccases in cells were examined. The experimental results indicated that the mixed culture may immediately induce an increase in the content of H_2_O_2_ in cells (Fig. 6). Further experimental results showed that CAT and the plasma membrane NAD(P)H oxidase inhibitor DPI might not only inhibit the production of H_2_O_2_ induced by the mixed culture but also inhibit the acceleration of the synthesis of laccases from the mixed culture (Fig. 6). The above experimental results suggested that H_2_O_2_ is a signaling molecule involved in the synthesis of laccases in cells induced by the mixed culture.

### Effects of mixed culture on biomass, phenolics, and flavonoids in cells

**Figure 7:**
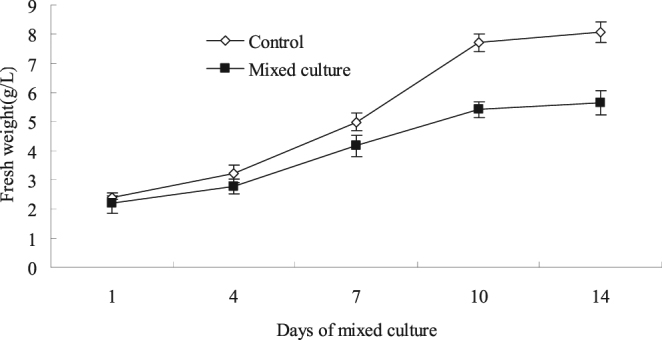
Effects of mixed culture on cell growth. The experimental material was mixed with 2.0% (v/v) *Phoma* sp. BZJ6 with *S*. *bambusicola* for 3 days. The control group was *S*. *bambusicola* and *Phoma* sp. BZJ6 cultured individually, the culture conditions were the same as those of the mixed culture. The results shown are the average of three independent experiments.

Figure 7 shows that the biomass of the cell suspension was not significantly affected on the fourth day after the mixed culture, and the biomass of the cells in the mixed culture was appreciably reduced by 30% of that in the control group from 10 days to 14 days. Results suggested that the mixed culture of *S*. *bambusicola* and *Phoma* sp. BZJ6 exhibited a negative regulatory effect on cell viability and biomass.

**Figure 8:**
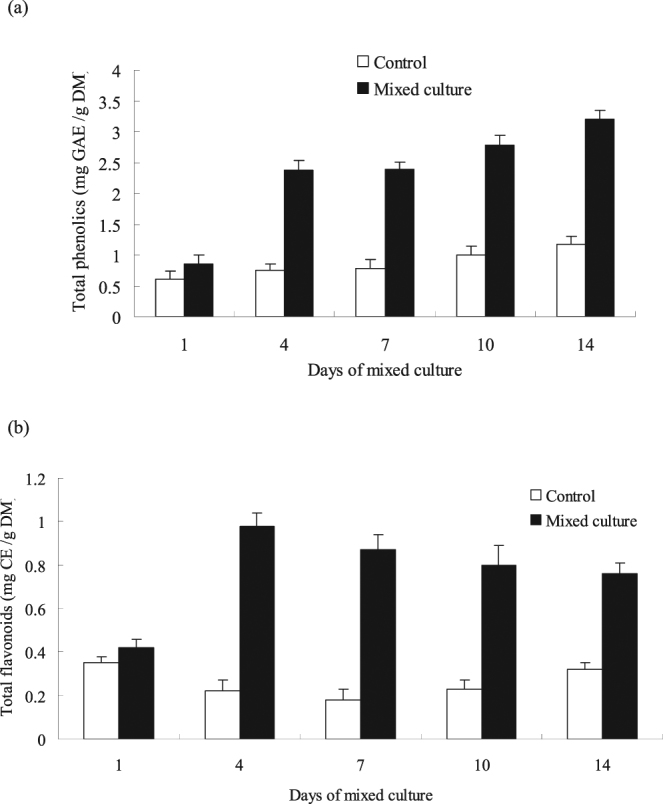
Effects of mixed culture on total phenolics (**a**) and total flavonoids (**b**) production. The experimental material was mixed with 2.0% (v/v) *Phoma* sp. BZJ6 with *S*. *bambusicola* for 3 days. The control group was *S*. *bambusicola* and *Phoma* sp. BZJ6 cultured individually, the culture conditions were the same as those of the mixed culture. The results shown are the average of three independent experiments.

Biological infection of plant tissues often causes changes in the metabolic activities of the biosynthetic pathway related to phenylpropanoids^31^. Thus, the task group also investigated numerous metabolites and enzymes related to the biosynthetic pathways. During the experiment, they were tested by mixed and non-mixed cultures. The effects of the mixed culture on the accumulation of total phenolics in the cell suspension are shown in Fig. 8a. Compared with those of the control group (non-mixed culture), the contents of total phenolics in cells in the mixed culture appreciably increased from day 1 to day 14. On the fourth day after the mixed culture, the yield of total phenolics increased by 3.2-fold compared with that of the control group and maintained steady growth after a long period of induction. Then, the contents of total phenolics increased by 2.7-fold on the 14th day after the mixed culture. Compared with that of the control group, the total flavonoids yield in the mixed culture greatly increased from day 4 to day 14. The total flavonoids yield increased by 4.5-fold on day 4, peaked, and then slightly decreased (Fig. 8b). However, the yield appreciably increased compared with that of the control group. Current studies show that the mixed culture may not only activate an increase in the laccase yield but also effective strategy to enhance the production of different active metabolites.

### Effects of mixed culture on the activity of phenylalanine ammonia lyase (PAL) and chalcone isomerase (CHI) in the cells

**Figure 9:**
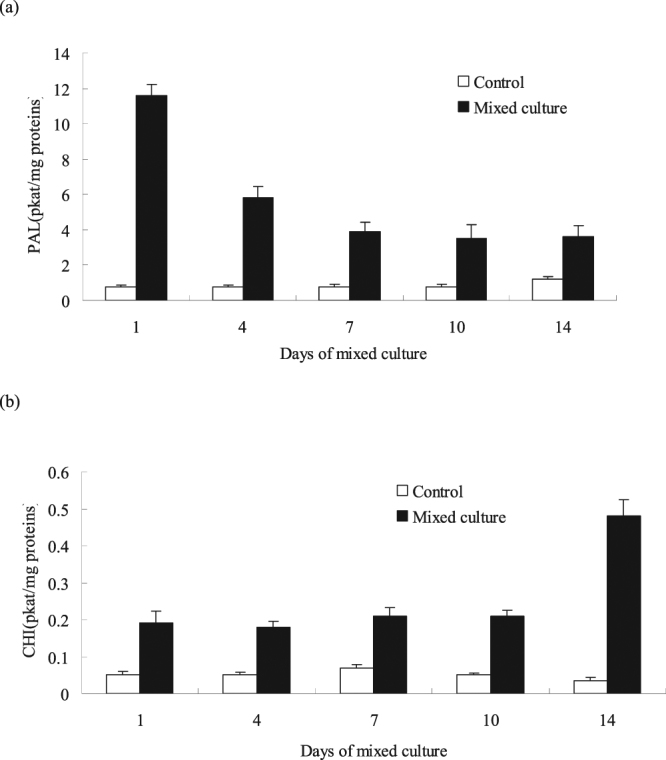
Effects of mixed culture on the activity of PAL (**a**) and CHI (**b**). The experimental material was mixed with 2.0% (v/v) *Phoma* sp. BZJ6 with *S*. *bambusicola* for 3 days. The control group was *S*. *bambusicola* and *Phoma* sp. BZJ6 cultured individually, the culture conditions were the same as those of the mixed culture. The results shown are the average of three independent experiments.

After the mixed culture, the activities of the key enzymes PAL and CHI related to the phenylpropanoid/flavonoid pathways were tested. In the cells after the mixed culture, the activities of PAL and CHI (Fig. 9) were highly induced, and the induction level depended upon the duration of the mixed culture. After the mixed culture, the PAL activity in the cell suspension was enhanced and peaked on day 1, approximately 15 times that of the control group. Thereafter, the PAL activity gradually decreased, but a significant increase occurred. Until day 14, the activity increased by 3-fold compared with that of the control group. Results suggested that during the mixed culture, the CHI enzyme activity in the cells increased, and the CHI activity increased by 14-fold under the stimulation of the mixed culture on day 14 compared with that of the control group. Furthermore, the phenylpropanoid and laccase yields in the mixed cells were positively correlated with the PAL and CHI enzyme activity.

## Discussion

Traditionally, the syntheses of most fungal laccases require induction by phenolics compounds and/or heavy metal ions. However, these poisonous elicitors may pollute the environment; thus, traditional laccase production methods are expensive and unsafe^32^. The production of laccases through mixed fermentation has been developed in recent years^20^. This process is characterized by simple operations that markedly improved the synthesis of laccases without requiring extra additives and has recently become a developmental trend in the research field of laccases. However, not all fungi may increase the laccase yield from the mixed culture^18,21,33–35^. *S*. *bambusicola* and endophytic fungus from *B*. *densiflorum* in the long term coexistence form a complex and special relationship that is likely a symbiotic or competitive relation or may vary with a variety of factors^36^. By taking the special advantages of environmental factors, the task group selected *B*. *densiflorum* producing *S*. *bambusicola* as the material for bio-separation in the experiment. A total of 36 strains of endophytic fungi were isolated from *B*. *densiflorum*, the host of *S*. *bambusicola*. However, not all endophytic fungi might increase the laccase yield in the mixed culture with *S*. *bambusicola*. The task group added *S*. *bambusicola* in the mixed culture with some other endophytic fungi from *B*. *densiflorum*, but the results showed that enzyme activity was not high, and the mixed culture retarded the growth and even stopped the growth few days later. The syngenetic process or trophism among the different fungal species is necessary for mixed fermentation, and the adverse effects of large intermediate products must be overcome. Enzymatic activity greatly improved after different strains were combined^37^. Liquid culture was used to determine whether the mixed culture may promote the synthesis of laccases, and the strain BZJ6 that had the best promoting effect was screened from the 36 strains of endophytic fungi. The results showed the significant effect of the mixed culture of *S*. *bambusicola* and *Phoma* sp. BZJ6 on laccase production, the co-culture of laccase-producing strains, and other strains exhibited high performance, are environmentally friendly, and involve low-cost methods for laccase production. Mixed fermentation utilized the different effects of various strains, and mutualistic effect was observed among the mixed strains. A highly appropriate enzyme ratio results in higher enzymatic production capacity of the mixed strains compared with that of a single strain, which leads to increased output.

The laccase activity in the co-culture system was 9.18-fold higher than that in the pure culture, which indicates that the mixed culture is an effective method to increase laccase yield. Previous report suggested that a fungus, after the co-culture with other microbes, may increase the laccase yield. Researchers generally believe that an increase in the laccase yield may be manifested as a defense response to another microbe^18^. The experimental results showed that the mixed culture of *S*. *bambusicola* and *Phoma* sp. BZJ6 induces the synthesis of laccases in *S*. *bambusicola*. The mixed culture can induce a variety of cell responses, including the synthesis of secondary metabolites^38,39^. However, molecular mechanisms, such as signal transduction in the synthesis of secondary metabolites, in the mixed culture remain unknown. Nevertheless, a few studies on the adversity and stress induced by pathogens and elicitors exist. The mixed culture is seen as an extracellular stimulus. First, it stimulates the release of a substance from cells that recognize and bind to specific receptors on the cell membrane. Consequently, the cells produce specific intracellular messenger substances that regulate the expression of related genes in the nucleus via corresponding signal transduction pathways^40^. Eventually, the defensive secondary metabolic system in cells is activated to promote the synthesis of secondary metabolites. In addition to the accumulation of nitric oxide, the production of ROS by oxidation leads to another characteristic reaction of cells that occur generally under various stress conditions.

Studies have found that exogenous NO produces oxidative stress and induces apoptosis through the calcium signaling pathway and triterpenoid biosynthesis that increase intracellular ROS levels^41^. Brassica plants can tolerate zinc; the metabolism of ROS and active nitrogen in roots were significantly changed, and the production of ROS and activation of nitrogen metabolism are stress responses. However, the oxidative stress component is more dominant than the nitrification stress^42^. The experimental results demonstrated that the mixed culture induced laccase biosynthesis in cells and production of NO and ROS (Figs 3 and 5), indicating that the mixed culture can activate signal transduction events leading to NO and ROS production in cells (*S*. *bambusicola* and *Phoma* sp. BZJ6). The mixed cultures of cells were treated by the NO specificity quencher cPITO, nitric oxide synthase inhibitor PBITU, plasma membrane NAD(P)H oxidase inhibitor DPI, as well as the O_2_^−^ and H_2_O_2_ quenchers SOD and CAT. Results indicated that cPITO and DPI not only inhibited NO synthesis and ROS accumulation in the cells but also inhibited the promotion of laccase synthesis in the mixed culture, indicating that NO, O_2_^−^, and H_2_O_2_ were the signals involved in the regulation of laccase synthesis in the mixed culture. cPITO and PBITU also partially inhibited the induction of oxidative burst in the mixed culture. The experimental results confirmed that NO, O_2_^−^, and H_2_O_2_ are three kinds of signaling molecules that involve the synthesis and accumulation of laccases induced by the mixed culture. Under normal circumstances, the physiological activities of cells often require the common participation of a variety of signaling molecules, and the biological cross-talk among different signaling molecules is a highly general phenomenon in cellular signal transduction^43^. However, the mechanisms of cross-talk for most signaling molecules remain unclear. Recent studies showed that NO and H_2_O_2_ pathways may involve the cytosolic alkalization-mediated signal transduction of darkness-induced stomatal closure in *Vicia faba* cells^44^; high concentrations of hydrogen sulfide inhibits root growth by stimulating the development of a series of signal transduction pathways, including ROS accumulation, activation of mitogen-activated protein kinase 6, and production of NO^45^. However, the mechanism by which NO and ROS mediate the mixed culture to induce the synthesis and accumulation of secondary metabolites of cells needs further study.

The mixed culture of *S*. *bambusicola* and *Phoma* sp. BZJ6 exhibited a negative regulatory effect on cell viability and biomass, and the inhibition that activates cell growth is considered to be caused by metabolic flux shunt, that is, the secondary metabolism is more active than the primary metabolism and even negatively related to cell growth and production of secondary metabolites^46^. However, the task group also found that polysaccharide extracts from the fungus *Trametes* sp. TT1 in the rhizosphere soil from the plant bamboo may increase the biomass of *S*. *bambusicola* ^47^. This finding was also observed in many studies on hyphae or extracts from plant cells, which indicates that the biological responses of *S*. *bambusicola* to different fungi or fungal extracts may vary with cell lines and culture conditions.

Biological infections of plant tissues often cause changes in the metabolic activities of the biosynthetic pathway related to phenylpropanoids^31^. Thus, the task group also investigated several metabolites and enzymes related to the biosynthetic pathways. Current studies show that the mixed culture may not only increase laccase yield but also effectively enhance different phenolics compounds. The mixed culture may cause the accumulation of large amounts of total phenolics and total flavonoids in cells probably because *S*. *bambusicola* and endophytic fungus *Phoma* sp. BZJ6, after being mixed, may activate substances produced in the defense reactions of the strains. For example, total phenolics and total flavonoids were heavily accumulated in the cells of *Hypericum perforatum* under the induction of the fungus *Nomuraea rileyi* ^48^. Therefore, an increase in the phenylpropanoid yield may be caused by the extracellular products released from thalli that activate the cell defense pathways. We observed that the mixed culture is most likely to have complex interactions with the signal transduction induced by its unique recognition pattern, thereby resulting in plant defense responses.

Current studies also showed that the mixed culture induced the PAL and CHI enzyme activities, and the rapid activation of PAL was related to the presence of a broad range of active compounds in mixed cells, including proteins, glycoprotein, fat, and free oligosaccharides, that trigger early plant defense responses^49,50^. In the cell culture induced by several different fungi, the results indicated an increase in the PAL activity, PAL is a key regulatory enzyme that leads to the widespread formation of phenylpropane metabolites^51^, whereas CHI is an active enzyme for the biosynthesis of flavonoids^52^. Thus, the contact between different strains induced by the mixed culture and the permeation of active substances may effectively trigger the PAL/CHI enzyme activity. In addition, the changes in the yield of phenolics and total flavonoids may be a manifestation of such changes in the enzyme activity. In the present study, both the phenylpropanoid yield and the PAL/CHI enzyme activity were increased, suggesting that the mixed culture may not only stimulate an increase in the laccase yield but also effectively increase the yield of phenolics.

## Conclusions

In summary, this study suggested that the mixed culture of *S*. *bambusicola* and the endophytic fungus *Phoma* sp. BZJ6 stimulated the cells to significantly enhance the laccase yield. The laccase yield peaked when the main constituents of the culture medium were soluble starch, yeast extract, and 2.0% (v/v) *Phoma* sp. BZJ6. Thus, the mixed culture is suitable for increasing the laccase yield in a short incubation time for commercial cell culture processes. Moreover, this system offers a promising way to study the biosynthetic pathway of secondary metabolites in cells. Furthermore, the regulation for the mixed culture was also investigated. After using the mixed culture, a large quantity of signaling molecules of NO and ROS were synthesized in cells, resulting in reduced biomass and increased synthesis of phenolics and flavonoids. Thus, these compounds are important components of the cell reaction system induced by the mixed culture. In the cells produced in the mixed culture, oxidation burst and ROS synthesis were inhibited by the NO quencher cPITO. The oxidative inhibitor DPI and H_2_O_2_ quencher SOD inhibited the promotion of NO in cells during laccase synthesis, indicating that NO is partially dependent on the oxidation pathway to promote cell laccase biosynthesis. In this perspective, further exploration is needed on the degree of NO dependence on bursting and the ROS signaling pathways, as well as other signaling pathways in the induction of laccase synthesis in the mixed culture, to explain the complexity of secondary metabolite synthesis and signal transduction mechanism in cells. Research showed that the heavily accumulated phenylpropanoids and flavonoids were highly associated with the PAL and CHI activities. In future studies, relationships between laccase and total phenolics/total flavonoids/signaling molecules in the cells after the mixed culture should be investigated to elucidate the possible mechanism. Such information could provide technical and theoretical bases for the usage of bio-activators from *S*. *bambusicola* in food and pharmaceutical industries. Therefore, we recommend that the production of secondary metabolites of *S*. *bambusicola* could be achieved by endophytic fungi that induce partial modification of the regulation of the metabolites. Moreover, the mixed culture could be applied for production methods that utilize laccase as a key activator.

## Materials and Methods

### Strains, chemicals and culture medium

*S*. *bambusicola* GZ-13 × 1 was collected and identified by the Institute of Fungal Resources, Guizhou University, China. Two kinds of fungi (*S*. *bambusicola* and *Phoma* sp. BZJ6) were cultured in potato dextrose agar (PDA) for 7 days and transferred into a 500 mL triangular flask containing 150 mL of basal medium for shaking culture at 26 °C and 120 r/min. The basal medium was made from 15 g/L glucose, 1.5 g/L sodium nitrate, 1.0 g/L KH_2_PO_4_, 0.5 g/L MgSO_4_·7H_2_O, 0.1 g/L Na_2_HPO_4_·5H_2_O, 0.01 g/L CaCl_2_, 1 mg g/L FeSO_4_·7H_2_O, 2 mg CuSO_4_·5H_2_O.

Carboxy-2-phenyl-4,4,5,5-tetramethylimidazoline-1-oxyl-3-oxide (cPITO), aminoguanidine (AG), 2,2′-azinobis-(3-ethylbenzthiazoline-6-sulphonate) (ABTS) were purchased from Sigma-Aldrich (St. Louis, MO, USA). All other chemicals and reagents were analytical-grade.

### Separation and screening of endophytic fungi from *B*. *densiflorum*

The young shoots and leaves of fresh *B*. *densiflorum* were obtained, rinsed with tap water, and naturally dried on a filter paper. The young shoots and leaves were cut into approximately 0.5 cm segments. The surfaces were disinfected by soaking and washing in 75% alcohol for 2–3 min, rinsing 4–6 times with aquae sterilisata, disinfecting with 0.1% mercury bichloride for 3–5 min, rinsing 4–6 times with aquae sterilisata, and cutting into small pieces with a sterile knife^53^. The pieces were separately placed into the PDA containing penicillin, cultured at 26 °C, purified, and then stored for use. By the co-culture of *S*. *bambusicola* and different endophytic fungi isolated from *B*. *densiflorum*, the strains that might promote *S*. *bambusicola* to produce laccases were identified.

### Determining laccase activity

Laccase activity was determined by monitoring the absorbance change at 420 nm related to the rate of oxidation of 1 mM ABTS to its cation radical in 25 mM Na-acetate buffer (pH 3.8) at room temperature^54^. One unit of enzyme activity was defined as the amount of enzyme required to oxidize 1 μmol ABTS per minute. All experiments were performed using at least three replicates. The data presented correspond to mean values, the standard deviation being lower than 15%.

### Strain identification

#### Morphological observation

The mycelia were inoculated in PDA, and the colony characteristics were cultured at 26 °C and observed. The size and growth of thallospore were observed under a microscope.

### ITS sequence-based amplification and analysis

Strains were inoculated in PDA for 4-day shaking culture at 26 °C. The mycelia were collected by centrifugation and the total DNAs were extracted from the thalli with the Genomic DNA Kit. The rDNA-ITS universal primers (ITS-1, ITS-4) were used for PCR amplification with the total DNA as the templates, and the PCR products after purification were sampled and delivered for determining the DNA sequence. The obtained ITS sequences were analyzed by Blaster in contrast to the nucleic acid database sequences in the GenBank. ITS-1: 5′-GTAGGTGAACCTGCGG-3′, ITS-4: 5′-TCCTCCGCTTATTGATATGC-3′; PCR procedures included pre-degeneration for 5 min at 95 °C, degeneration for 1 min at 95 °C, renaturation at 50 °C, and prolongation at 72 °C, a total of 35 cycles, and final prolongation for 10 min at 72 °C^55^. Primer synthesis and sequencing were completed by Sangon Biotech Co., Ltd. (Shanghai, China).

### Mixed culture

Glucose in the basal fermentation medium was separately replaced with equal amounts of sucrose, fructose, and soluble starch to choose the best carbon source. Then, the sodium nitrate in the basal fermentation medium was separately replaced with equal amounts of beef extract, yeast extract, peptone, and ammonium nitrate to choose the best nitrogen source. *S*. *bambusicola* cultured for 3 days, together with *Phoma* sp. BZJ6, a endophytic fungus from *B*. *densiflorum*, were added to the culture medium in the flask by 1%–4% of the inoculation amount to choose the optimal inoculation amount. In this study, spores and mycelia were scraped from the PDA tablet surface and beaten using sterile glass beads, diluted 10^7^ spores per mL with aquae sterilisata, inoculated in a triangular flask containing the fermentation medium, shaken at 26 °C and 120 r/min, and cultured for 4 days. The mycelium pellets were placed into a sterile mixer and centrifuged for 10 s at 3000 rpm, forming the homogeneous mycelia for vaccination. The homogeneous *S*. *bambusicola* was added to the optimized and basal media by 20% of the inoculation amount. The *Phoma* sp. BZJ6, a homogeneous endophytic fungus from *B*. *densiflorum*, was added by 1–4% of the inoculation amount to the *S*. *bambusicola*, medium cultured for 3 days, and recultured for 1–14 days.

### Determining the NO, H_2_O_2_ and O_2_^−^ contents in mycelia

The intracellular NO content was determined by the method described in the reagent kit provided by Beyotime Biotechnology. 10 mL broth was taken and centrifuged, and the precipitate was ground with a small amount of quartz sand. The grinding fluid was rinsed with 450 mL HEPE (pH 7.2) buffer solution and centrifuged for 5 min at 11 000 r·min^−1^. The supernatant was taken and determined by the method in the kit, and the NO concentration was calculated by the given formula. The intracellular hydrogen peroxide content was determined by the method in the reagent kit provided by Beyotime Biotechnology.

To examine the O_2_^−^ production, 150 μmol/L of tetranitroblue tetrazolium chloride (NBT) and 468 μmol/L of nicotinamide adenine dinucleotide disodium solution were added in a test tube, and the different of sample solutions were added. The reaction was started by adding 60 μmol/L of 5-methylphenazinium methosulfate, and the absorbance was measured at 530 nm at 25 °C after 5 min of reaction. The cell culture medium without the mixed culture was used as control^56^.

### Determining biomass

The fermented mash was filtered through a funnel, with the filtrate removed. The collected thalli were washed with distilled water, and after the removal of the supernatant, the thalli were dried to a constant weight at 35 °C and weighed.

### Determining the total phenolics content in mycelia

The scraped mycelia were extracted for 10 min with the alcohol-acetone solution (1:1) in water bath by ultrasonic method at room temperature and centrifuged at 4,800 r/min, and the supernatant was collected and sampled with the right amount of Folin–Ciocalteu reagents and 20% sodium carbonate solution, evenly mixed and placed quietly for 40 min. The absorbance was determined with the wavelength of 725 nm. The results were expressed as milligrams of gallic acid equivalents (GAE) per gram of dry mass (mg GAE/g DM)^57^.

### Determining the total flavonoids content in mycelia

Ethanol solution (60%) was added to the scraped mycelia by the solid-liquid ratio of 1:15, and the mycelia were extracted for 1 h at 80 °C. The filtrate after the repeated extraction and filtration was added to the chromatographic column of macroporous resin AB-8 for adsorption, and with impurities (such as pigments) scoured off with distilled water until a colorless effluent was discharged, and eluted with 80% ethanol solution. Then, a concentrated eluent was collected and sampled. The sampled eluent with 5% NaNO_2_, 10% Al(NO_3_)_3_, and 1 mol/L NaOH added was shaken up and placed for 30 min, with the absorbance determined with the wavelength of 510 nm. The results were expressed as milligrams of catechin equivalents (CE) per gram of dry mass (mg CE/g DM)^58^.

### Enzyme extraction and assays

The extraction procedure for determination of antioxidant enzyme assays was based on the method as previously described by Gadzovska *et al*.^59^. The enzyme extract was prepared by homogenizing 1 g of frozen sample in 2 mL 0.1 M KH_2_PO_4_/K_2_HPO_4_ buffer at pH 8.0, containing 2 mM ethylenediamine tetra-acetic acid (EDTA), 1.4 mM β-mercaptoethanol and 1% (w/v) polyvinylpyrrolidone (PVP). The homogenate was centrifuged at 13,000 rpm for 20 min at 4 °C. The supernatant was collected for determination of protein content and enzyme assays. Protein contents in enzyme extracts were performed with a Bio-Rad Protein Assay Reagent using bovine serum albumin as a standard^60^.

PAL was assayed in 50 mM Tris–HCl at buffer pH 8.8 containing 2% (w/v) solution of L-phenylalanine and enzyme extract. Enzyme assay mixtures were incubated at 40 °C for 60 min. PAL activity was determined by measuring the rate of formation of trans-cinnamic acid as increase in absorbance at 290 nm. The PAL activity was expressed in pkat/mg proteins^61^.

CHI was assayed in 60 mM KH_2_PO_4_/K_2_HPO_4_ buffer at pH 8.0, containing 50 mM KCN to inhibit POD activity. Reaction was initiated by mixing enzyme extract and 2′4,4′,6-tetrahydroxychalcone. Enzyme assay mixture was incubated at 30 °C for 45 min. The kinetics of the reaction was monitored by measuring the decrease in absorbance at 400 nm. The CHI activity was expressed in pkat/mg proteins^48^.
